# Hierarchical and coupling model of factors influencing vessel traffic flow

**DOI:** 10.1371/journal.pone.0175840

**Published:** 2017-04-17

**Authors:** Zhao Liu, Jingxian Liu, Huanhuan Li, Zongzhi Li, Zhirong Tan, Ryan Wen Liu, Yi Liu

**Affiliations:** 1 Hubei Key Laboratory of Inland Shipping Technology, School of Navigation, Wuhan University of Technology, Wuhan, China; 2 National Engineering Research Center for Water Transport Safety, Wuhan, China; 3 Department of Civil, Architectural and Environmental Engineering, Illinois Institute of Technology, Chicago, Illinois, United States of America; Beihang University, CHINA

## Abstract

Understanding the characteristics of vessel traffic flow is crucial in maintaining navigation safety, efficiency, and overall waterway transportation management. Factors influencing vessel traffic flow possess diverse features such as hierarchy, uncertainty, nonlinearity, complexity, and interdependency. To reveal the impact mechanism of the factors influencing vessel traffic flow, a hierarchical model and a coupling model are proposed in this study based on the interpretative structural modeling method. The hierarchical model explains the hierarchies and relationships of the factors using a graph. The coupling model provides a quantitative method that explores interaction effects of factors using a coupling coefficient. The coupling coefficient is obtained by determining the quantitative indicators of the factors and their weights. Thereafter, the data obtained from Port of Tianjin is used to verify the proposed coupling model. The results show that the hierarchical model of the factors influencing vessel traffic flow can explain the level, structure, and interaction effect of the factors; the coupling model is efficient in analyzing factors influencing traffic volumes. The proposed method can be used for analyzing increases in vessel traffic flow in waterway transportation system.

## Introduction

With the rapid development of water-transportation systems and the innovation of shipbuilding technology, the characteristics of vessel traffic flow including number, type, size, and cruising speed of vessels constantly change. In addition, the characteristics of vessel traffic flow are becoming increasingly complex [1]. This complexity increases the safety risk of waterway traffic and mismatching pressure between waterway transportation demand and limited capacity of navigable waterways. Hence, waterway traffic safety monitoring and navigation scheduling efficiency need to be improved. The factors that influence vessel traffic flow have features of hierarchy, uncertainty, nonlinearity, complexity, etc. Understanding the characteristics of vessel traffic flow are crucial to maintain safe and efficient operations of waterway transportation. Therefore, it is significant to clarify the impact mechanism of such influencing factors. Several previous studies have analyzed characteristics of vessel traffic flow and identified a list of influencing factors, creating some basis for analyzing the impact mechanism of influencing factors.

**Fig 1:**
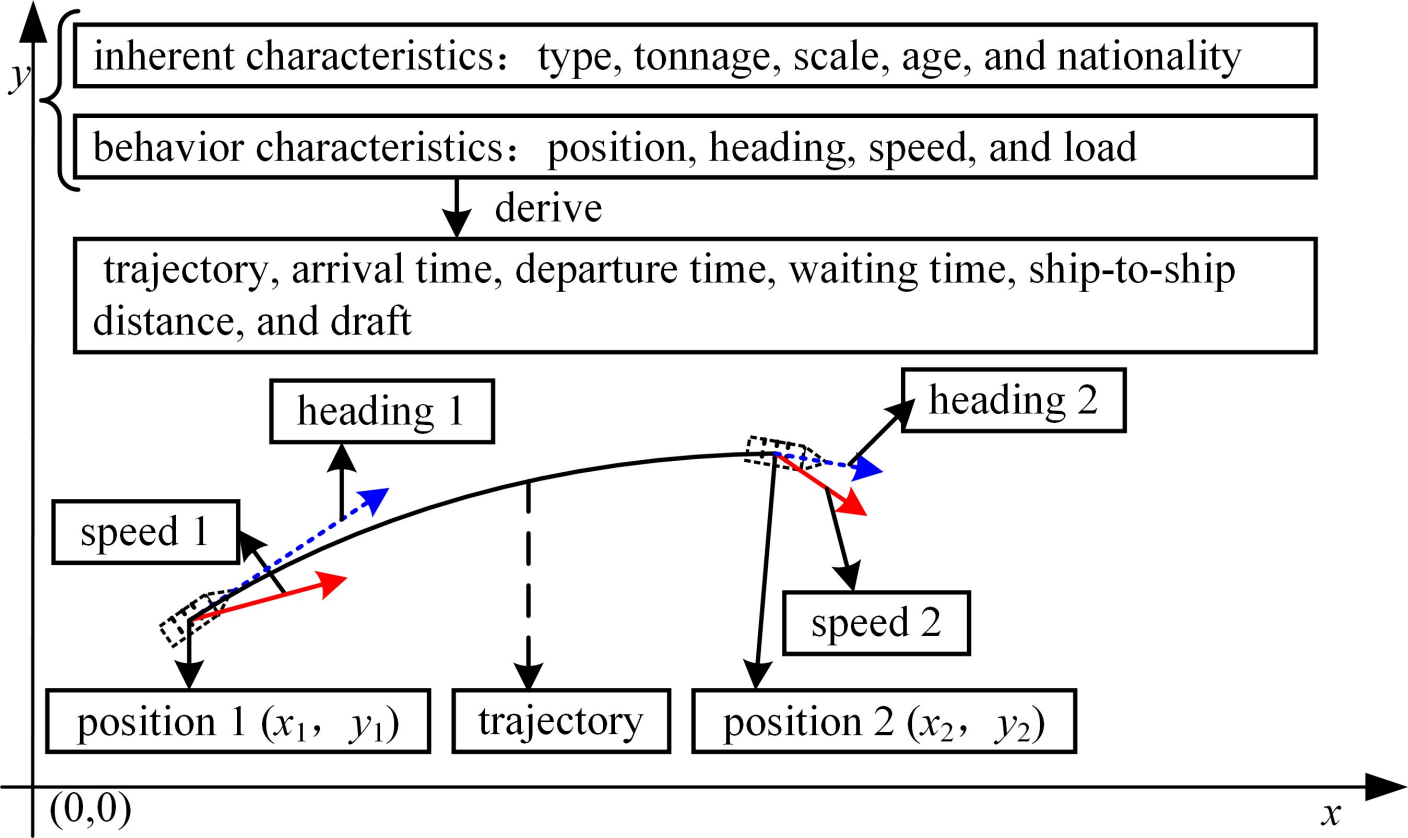
Elements of Vessel Traffic Flow.

Vessel traffic flow generally contains five basic elements: the location, direction, width, density, and velocity of traffic flow. In general, data obtained by radar and Automatic Identification System (AIS) is analyzed to study vessel traffic flow [2–5]. For simulation-based collision risk analysis, the attributes considered for a vessel traffic event mainly include ship particulars (i.e., type, length, and width), route, departure time, and speed [6]. In the use of simulation techniques based on integrated bridge system and cellular automata to model vessel traffic flow, the identity, type, position, course, speed, and navigation status of a ship are considered [7]. A ship has inherent characteristics (type, tonnage, scale, age, and nationality, etc.) and behavior characteristics (position, heading, speed, and load, etc.) when it sails in waterways [8, 9]. The trajectory, arrival time, departure time, waiting time, and ship-to-ship distance are derived from the change in the behavior of ships in time series. In addition, the draft is derived from the load of a ship. Therefore, elements of vessel traffic flow include, but are not limited to, traffic volume, type, tonnage, scale, age, nationality, position, trajectory, arrival time, departure time, speed, heading, ship-to-ship distance, draft, and their statistical distribution and representative values (i.e., mean, minimum, and maximum values). Fig 1 shows the elements of vessel traffic flow.

**Fig 2:**
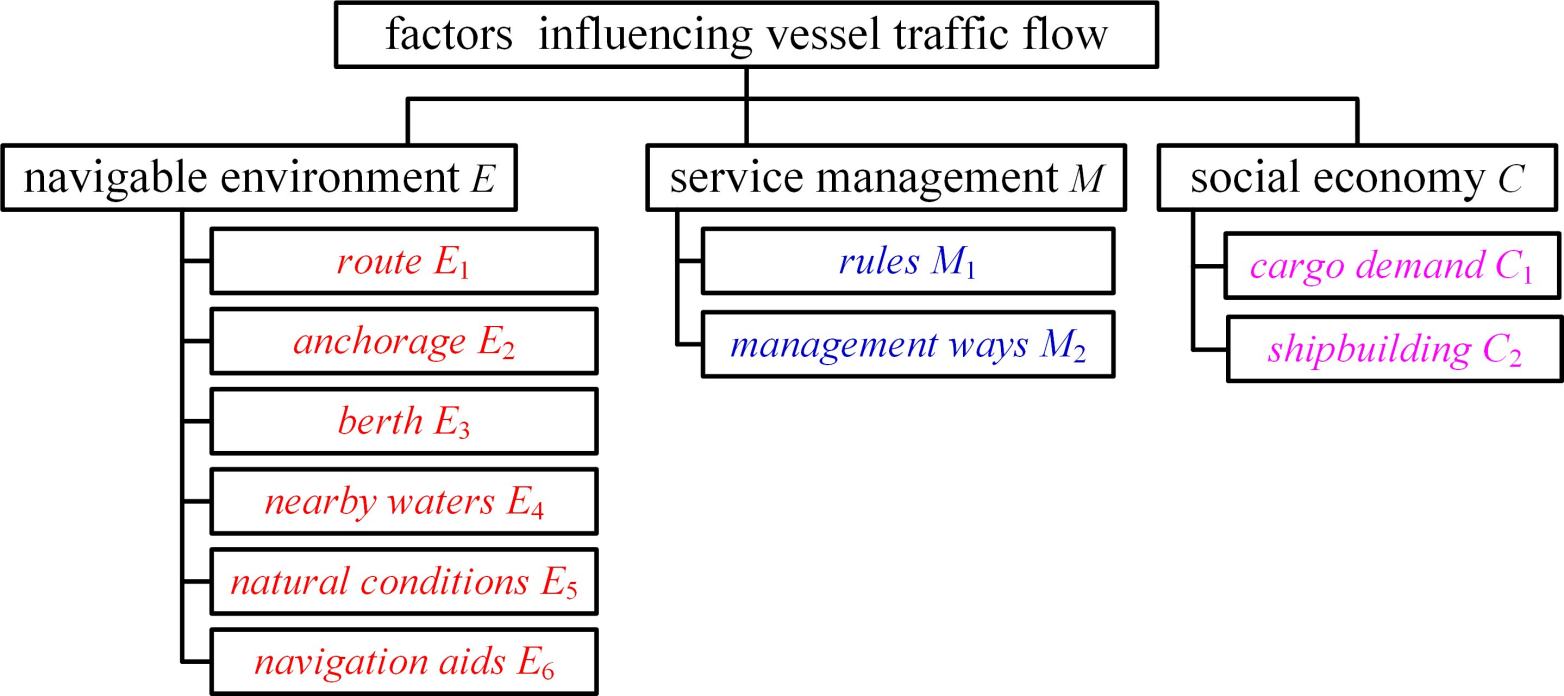
Factors Influencing Vessel Traffic Flow.

**Fig 3:**
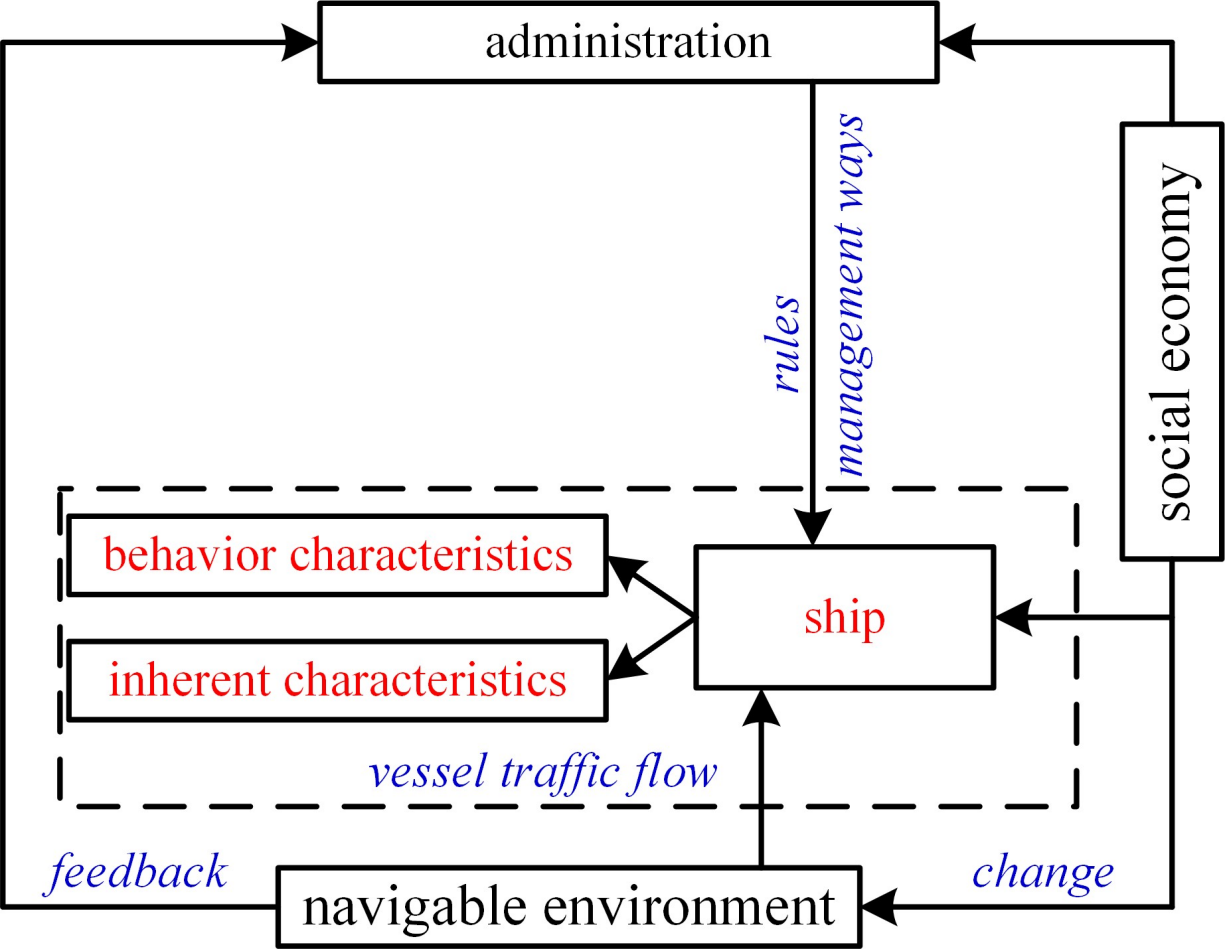
Relationship of Factors Influencing Vessel Traffic Flow.

The factors that influence vessel traffic flow have been used to evaluate water traffic safety, predict vessel traffic volume, perform simulation-based analysis, and conduct modeling of waterway transportation system [10–13]. In a previous research documented in [14], factors influencing vessel traffic flow are divided into ship and environment the two categories which include ship, natural environment (i.e., wind, water flow, and tide, and rainfall), functional waters (i.e., berth, route, and anchorage), and service management. In the current study, the factors influencing vessel traffic flow are classified into the following three aspects: navigable environment, service management, and social economy. Figs 2 and 3 present the factors influencing vessel traffic flow and their relationship, respectively.

As illustrated in Fig 2, factors influencing vessel traffic flow attributable to the navigable environment include route, anchorage, berth, nearby waters, natural conditions, and navigation aids; factors associated with the service management include rules and management ways of waterway traffic; factors concerning the social economy include cargo demand and shipbuilding. Fig 3 depicts that vessel traffic flow is affected by factors in social economy, administration, and navigable environment contexts.

In previous studies, vessel traffic flow and its influencing factors are used to evaluate navigation safety, waterway transportation system, etc. The models of vessel traffic flow are studied systematically, and the factors influencing vessel traffic flow are distinguished. Particular to this study, factors that influence vessel traffic flow are categorized by using an interpretative structural modeling (ISM) model based on the relationships of those factors, and a coupling model is proposed to use quantitative indicators for the factors to reveal their impact mechanism.

The remainder of this paper is organized as follows: The next section describes the proposed model formulations. A hierarchical model is proposed based on the ISM model, and a coupling model that relies on weighting and quantitative indicators to analyze the relationship of factors influencing vessel traffic flow is introduced. The subsequent section focuses on analysis and verification. Specifically, the hierarchical model is employed to achieve hierarchical division of the factors, and the coupling model is validated using the traffic volume obtained from Port of Tianjin. The last section performs study summary and draws conclusions.

## Model formulation

**Fig 4:**
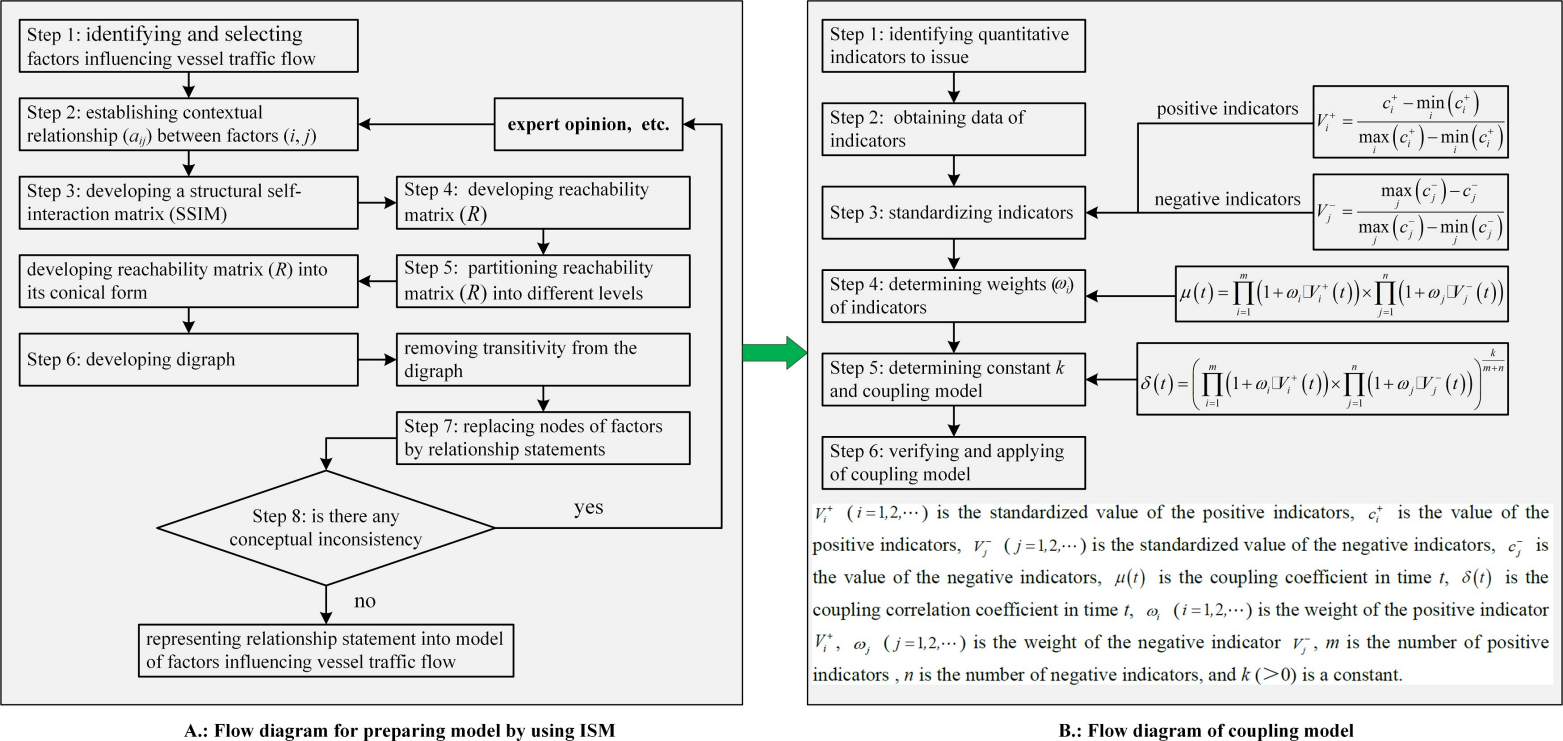
Flow Diagram of Model Formulation.

Fig 4 exhibits the procedure proposed for addressing the problem under investigation and model formulations. First, a hierarchical model is formulated. Then, a coupling model is introduced based on the hierarchical model of factors influencing vessel traffic flow. The values of model parameters are calibrated based on field data.

### The hierarchical model

The ISM method is an important structural modeling technology [15, 16]. It can effectively analyze the internal relationship between elements of complex systems, and ultimately construct a hierarchical structure model with multi-level recursive relations [17, 18]. The advantages of the ISM approach include depicting a graphical representation and a structured model of the original problem situation, which can be communicated more effectively to others. This will help develop a deeper understanding of the meaning and significance of a list of specified elements and the relationship among them [19–21]. The hierarchical modeling procedure has eight steps with Fig 4(A) showing the logical flow diagram.

Step 1: Identifying and selecting factors influencing vessel traffic flow

The first step in the structural modeling is to identify and select factors influencing vessel traffic flow based on previous studies and industry experts.

Step 2: Establishing contextual relationship (*a*_*ij*_) between factors (*i*, *j*)

It is crucial to state the contextual relationship between the factors for developing the structural model. Such a relationship can be determined by a brainstorming session, expert opinions, and method of systems analysis, etc. The contextual relationship can be written as follows:

$$a_{ij}=\left\{ \begin{array}{l} 1\begin{array}{cc} & S_{i}\overset{}{R}S_{j} \end{array}\\ 0\begin{array}{cc} & S_{i}\bar{R}S_{j} \end{array} \end{array}\right.$$ (1)

where *a*_*ij*_ is the contextual relationship between factors (*i*, *j*), *S*_*i*_ is factor *i*, *S*_*j*_ is factor *j*, **R** is the relation from factors *j* to *i* (i.e., factor *j* will influence factor *i*), and $\bar{R}$ denotes that no relation exists between factors *j* and *i* (i.e., factors *i* and *j* are unrelated).

Step 3: Developing a structural self-interaction matrix (SSIM)

Based on the contextual relationship (*a*_*ij*_) between factors (*i*, *j*), the SSIM can be developed. The SSIM belongs to the Boolean matrix, the elements of which are either 1 or 0. The main calculation rules of the elements in the SSIM include 0 + 0 = 0,0 + 1 = 1,1 + 0 = 1,1 + 1 = 1,0 × 0 = 0,0 × 1 = 0,1 × 0 = 0,1 × 1 = 1.

Step 4: Developing reachability matrix (*R*)

In the fourth step, a reachability matrix is developed from the SSIM. The reachability matrix is formed to describe the reachable degree of the nodes of a connected graph. The antecedent set shows the set of column elements, which corresponds to a row element of 1 of the reachability matrix. The SSIM is converted to the initial reachability matrix using the calculation rules of SSIM via Eq (2):

$$R={\left(I+A\right)}^{m-1}={\left(I+A\right)}^{m}=I+A+A^{2}+\cdots +A^{m-1}$$ (2)

where *A* is SSIM, *R* is the reachability matrix, and *I* is the identity matrix, *m* ≥ 2.

Step 5: Partitioning reachability matrix (*R*) into different levels

The reachability matrix includes the reachability and antecedent sets. The reachability set comprises of the factor itself and other factors that it may affect, whereas the antecedent set comprises the factor itself and the other factors that may affect it. Thereafter, the intersection set of the reachability and antecedent sets is derived for all factors. The levels of different factors are determined using Eq (3). For factors having the same level of reachability and intersection sets, they are listed at the top level of the model, and they do not affect other factors above their own level in the model. Once the top-level factor is identified, it is removed from consideration. Then, the same process is repeated to determine factors of the next lower level. This process is continued until the level of each factor is determined. Finally, all levels of the model are identified to develop the digraph.

$$C\left(S_{i}\right)=R\left(S_{i}\right)\cap Q\left(S_{i}\right)$$ (3)

where *C*(*S*_*i*_) is the intersection of the reachability and antecedent sets, *R*(*S*_*i*_) is the reachability set, *Q*(*S*_*i*_) is the antecedent set, and *S*_*i*_ is factor *i*.

Step 6: Developing digraph

The factors are listed in a digraph based on their levels by positioning the top-level factors at the top of the digraph, the second-level factors at second position, and so on. All levels are linked using a directed line. An initial structural model is then established. For displaying the relationship between the factors more intuitively and clearly, virtual nodes are increased in the initial structural model to obtain the final structural model.

Step 7: Replacing nodes of factors by relationship statements

The nodes of the factors are replaced by relationship statements herein.

Step 8: Checking for conceptual inconsistency

Finally, the conceptual inconsistency should be checked to ensure the credibility of the model.

### The coupling model

The coupling model of factors that influence vessel traffic flow is established based on the impact mechanism of the factors. The procedure of establishing the coupling model has six steps with Fig 4(B) showing the logical flow diagram.

Step 1: Identifying quantitative indicators to issue.

Due to complexity of vessel traffic flow affected by a large number of factors, the first step is to identify a vessel traffic flow issue. The issue could be associated with traffic volume, location, direction, width, density, or speed.

Step 2: Obtaining data of indicators

In the second step, field data on the indicators is obtained for quantitative analysis.

Step 3: Standardizing indicators

The quantifiable indicators need to be extracted and standardized before establishing the coupling model because of differences in their dimensionality and representation. The indicators can be divided into positive and negative indicators. A higher value of positive indicators implies a higher target value, whereas a higher value of negative indicators implies a smaller target value. After the indicators are standardized, the indicator will exhibit a value between 0 and 1. The standardization equations for the quantitative indicators are expressed in Eqs (4) and (5).

$$V_{i}^{+}=\frac{c_{i}^{+}-\underset{i}{\text{min}}\left(c_{i}^{+}\right)}{\underset{i}{\text{max}}\left(c_{i}^{+}\right)-\underset{i}{\text{min}}\left(c_{i}^{+}\right)}$$ (4)

where, $V_{i}^{+}$ (*i* = 1,2,⋯) is the standardized value of the positive indicators, and $c_{i}^{+}$ is the value of the positive indicators.

$$V_{j}^{-}=\frac{\underset{j}{\text{max}}\left(c_{j}^{-}\right)-c_{j}^{-}}{\underset{j}{\text{max}}\left(c_{j}^{-}\right)-\underset{j}{\text{min}}\left(c_{j}^{-}\right)}$$ (5)

where, $V_{j}^{-}$ (*j* = 1,2,⋯) is the standardized value of the negative indicators, and $c_{j}^{-}$ is the value of the negative indicators.

Step 4: Determining weights of indicators

For establishing a coupling model to capture the interaction effect of influencing factors, the coupling correlation coefficient is designed and is expressed in Eq (6).

$$\delta =f\left(V_{1}^{+},V_{2}^{+},\cdots ,V_{m}^{+};V_{1}^{-},V_{2}^{-},\cdots ,V_{n}^{-}\right)$$ (6)

where, *δ* is the coupling correlation coefficient, $V_{i}^{+}$ (*i* = 1,2,⋯) is the standardized value of the positive indicators, and $V_{j}^{-}$ (*j* = 1,2,⋯) is the standardized value of the negative indicators.

The degrees of impacts associated with the indicators are different. In this respect, a coupling coefficient is proposed to capture such differences, as shown in Eq (7). Further, a coupling correlation coefficient is defined by Eq (8).

$$\mu \left(t\right)=\prod_{i=1}^{m}\left(1+\omega_{i}\cdot V_{i}^{+}\left(t\right)\right)\times \prod_{j=1}^{n}\left(1+\omega_{j}\cdot V_{j}^{-}\left(t\right)\right)\;\mathrm{and}$$ (7)

$$\delta \left(t\right)=={\left(\mu \left(t\right)\right)}^{\frac{k}{m+n}}={\left(\prod_{i=1}^{m}\left(1+\omega_{i}\cdot V_{i}^{+}\left(t\right)\right)\times \prod_{j=1}^{n}\left(1+\omega_{j}\cdot V_{j}^{-}\left(t\right)\right)\right)}^{\frac{k}{m+n}}$$ (8)

where, *μ*(*t*) is the coupling coefficient at time *t*, *δ*(*t*) is the coupling correlation coefficient *t*, $V_{i}^{+}\left(t\right)$ (*i* = 1,2,⋯) is the standardized value of the positive indicator at time *t*, and $V_{j}^{-}$ (*j* = 1,2,⋯) is the standardized value of the negative indicator *t*. In addition, *ω*_*i*_ (*i* = 1,2,⋯) is the weight of the positive indicator $V_{i}^{+}$, *ω*_*j*_ (*j* = 1,2,⋯) is the weight of the negative indicator $V_{j}^{-}$, *m* is the number of positive indicators, *n* is the number of negative indicators, and *k* (>0) is a constant.

Prior to determining the coupling coefficient, relative weights of the indicators need to be determined by curve fitting the field data and performing a correlation analysis between the coupling coefficient and target value using Eq (7).

Step 5: Determining constant *k* and coupling model

In this step, the field data is applied to Eq (8) to determine the constant *k* in accordance with the best-fitting curve. Thereafter, the coupling correlation coefficient is calculated.

Step 6: Verifying and applying of the coupling model

Finally, the coupling correlation coefficient is used to validate the effectiveness of the coupling model for practical use subsequently.

## Model applications and validations

For applying and the proposed hierarchical model and coupling model, data on container ship flow in Port of Tianjin is collected. The dataset contains details of factors grouped by navigable environment (*E*), service management (*M*), and social economy (*C*) categories. Factors under navigable environment category include route (*E*_1_), anchorage (*E*_2_), berth (*E*_3_), nearby waters (*E*_4_), natural conditions (*E*_5_), and navigation aids (*E*_6_). Factors under service environment cover rules (*M*_1_) and management ways (*M*_2_). Factors under social economy are concerned with cargo demand (*C*_1_) and ship building (*C*_2_). With the historical data in place, the hierarchical model is first constructed to identify the key influential factors. This information is then used to validate the coupling model.

### Hierarchical model application

The application of the hierarchical model is to establish the structural self-interaction matrix collectively for factors under navigable environment, service management, and social economy categories that influence vessel traffic flow. Table 1 lists the structural self-interaction matrix *A*.

**Table 1:** SSIM (*A*) of Factors Influencing Vessel Traffic Flow.

	*E*_1_	*E*_2_	*E*_3_	*E*_4_	*E*_5_	*E*_6_	*M*_1_	*M*_2_	*C*_1_	*C*_2_
*E*_1_	0	0	0	0	0	1	1	1	0	0
*E*_2_	0	0	0	0	0	1	1	1	0	0
*E*_3_	1	1	0	0	0	1	1	1	0	0
*E*_4_	0	0	0	0	0	1	1	1	0	0
*E*_5_	1	1	1	1	0	1	1	1	0	0
*E*_6_	0	0	0	0	0	0	1	1	0	0
*M*_1_	0	0	0	0	0	0	0	0	0	0
*M*_2_	0	0	0	0	0	0	0	0	0	0
*C*_1_	0	0	1	0	0	0	0	0	0	0
*C*_2_	0	0	1	0	0	0	0	0	0	0

The next step is to create the reachability matrix using Eq (2). Table 2 presents the reachability matrix *R*.

**Table 2:** Reachability Matrix (*R*) of Factors Influencing Vessel Traffic Flow.

	*E*_1_	*E*_2_	*E*_3_	*E*_4_	*E*_5_	*E*_6_	*M*_1_	*M*_2_	*C*_1_	*C*_2_
*E*_1_	1	0	0	0	0	1	1	1	0	0
*E*_2_	0	1	0	0	0	1	1	1	0	0
*E*_3_	1	1	1	0	0	1	1	1	0	0
*E*_4_	0	0	0	1	0	1	1	1	0	0
*E*_5_	1	1	1	1	1	1	1	1	0	0
*E*_6_	0	0	0	0	0	1	1	1	0	0
*M*_1_	0	0	0	0	0	0	1	0	0	0
*M*_2_	0	0	0	0	0	0	0	1	0	0
*C*_1_	1	1	1	0	0	1	1	1	1	0
*C*_2_	1	1	1	0	0	1	1	1	0	1

With the structural self-interaction matrix and reachability matrix created, the influential factors can be partitioned. Without loss of generality, denoting the digraph of the hierarchical model as *G*_*n*_ = (*H*_*i*_,*E*_*k*_,*M*_*l*_,*C*_*m*_), (*n* = 1,2,3,⋯,20), *R*(*G*_*n*_) is the reachability set, *Q*(*G*_*n*_) is the antecedent set, and *C*(*G*_*n*_) is the intersection set of *R*(*G*_*n*_) and *Q*(*G*_*n*_). Table 3 shows the reachability, antecedent, and intersection sets of the first level. For instance, *R*(*G*_*n*_) = *R*(*G*_*n*_) = {*n*} (*n* = 7,8); hence, *G*_7_, *G*_8_ is at the top level. A new matrix is obtained by removing rows 7 and 8 and columns 7 and 8. The new reachability and antecedent sets of the new matrix are then obtained and employed to determine the factors at the second level, and so on. Finally, *G*_6_ is at the second level, *G*_1_, *G*_2_, and *G*_4_ are at the 3^rd^ level, *G*_3_ is at the 4^th^ level, and *G*_5_, *G*_9_, and *G*_10_ are at the 5^th^ level.

**Table 3:** Reachability, Antecedent, and Intersection Sets of the First Level.

*G*_*n*_	*R*(*G*_*n*_)	*Q*(*G*_*n*_)	*C*(*G*_*n*_)
1	1,6,7,8	1,3,5,9,10	1
2	2,6,7,8	2,3,5,9,10	2
3	1,2,3,6,7,8	3,5,9,10	3
4	4,6,7,8	4,5	4
5	1,2,3,4,5,6,7,8	5	5
6	6,7,8	1,2,3,4,5,6,9,10	6
**7**	**7**	**1,2,3,4,5,6,7,9,10**	**7**
8	**8**	**1,2,3,4,5,6,8,9,10**	**8**
9	1,2,3,6,7,8,9	9	9
10	1,2,3,6,7,8,10	10	10

**Fig 5:**
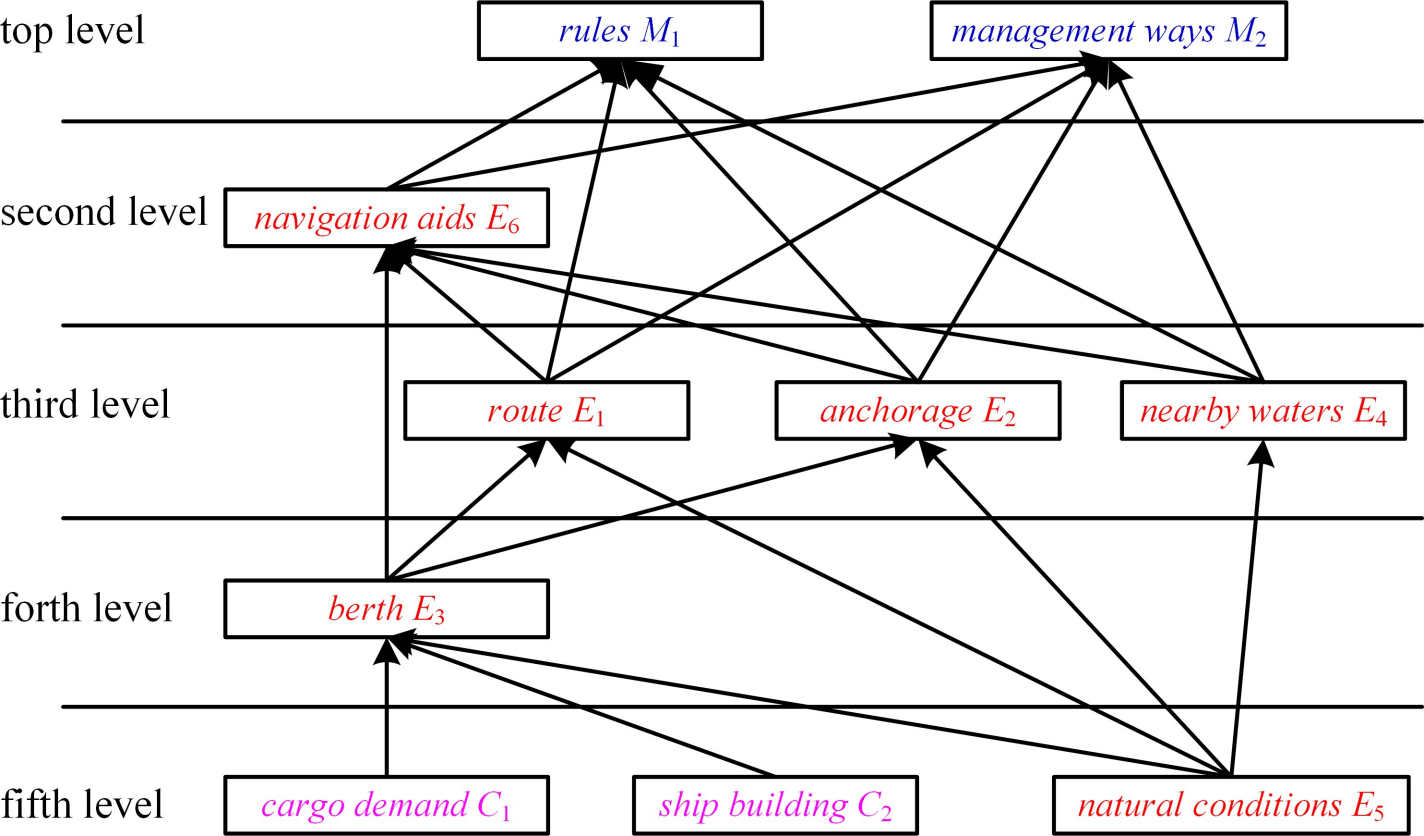
Initial Hierarchical Model of Factors Influencing Vessel Traffic Flow.

**Fig 6:**
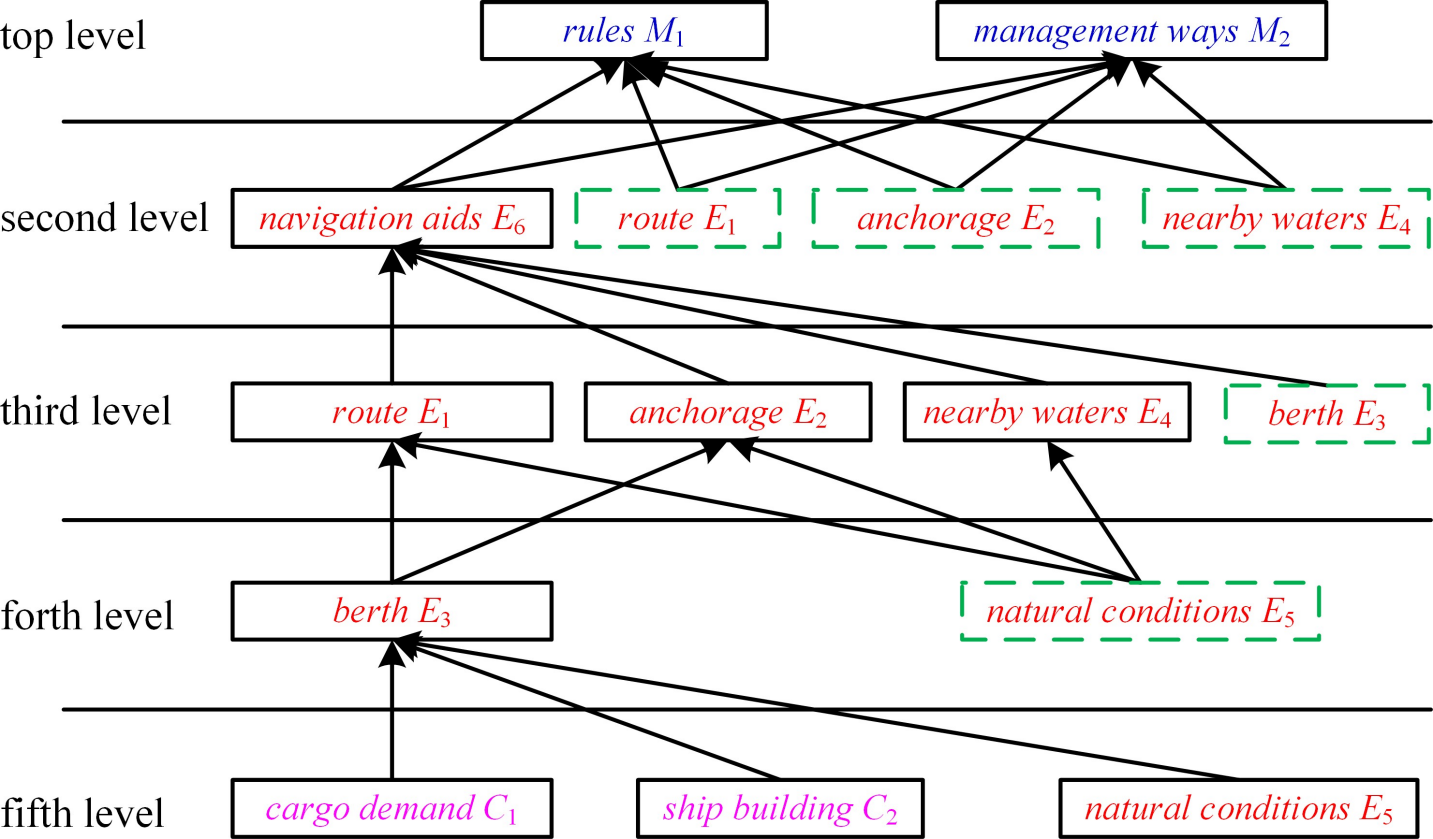
Hierarchical Model of Factors Influencing Vessel Traffic Flow.

Subsequently, the five levels of hierarchy can be used in conjunction with the structural self-interaction matrix can be employed to construct the hierarchical model. Fig 5 depicts the initial hierarchical model. For displaying the relationship between the factors more intuitively and clearly, the virtual nodes are increased in the initial hierarchical model to obtain the final hierarchical model, and the final hierarchical model is shown in Fig 6.

As shown in Fig 6, the cargo demand, shipbuilding (social economy), and natural conditions are factors at the bottom level. The berth is the factor at the forth level. The route, anchorage, and nearby waters are the factors at the third level. The navigation aids are the factors at the second level. The rules and management ways are the factors at the top level. The hierarchical model not only reflects factors directly affecting vessel traffic flow, but also factors influencing other factors that impact vessel traffic flow, as well as factors having complex interaction effects.

### Coupling model validation

To validate the coupling model, factors influencing vessel traffic volume are first identified based on the above hierarchical model.

**Fig 7:**
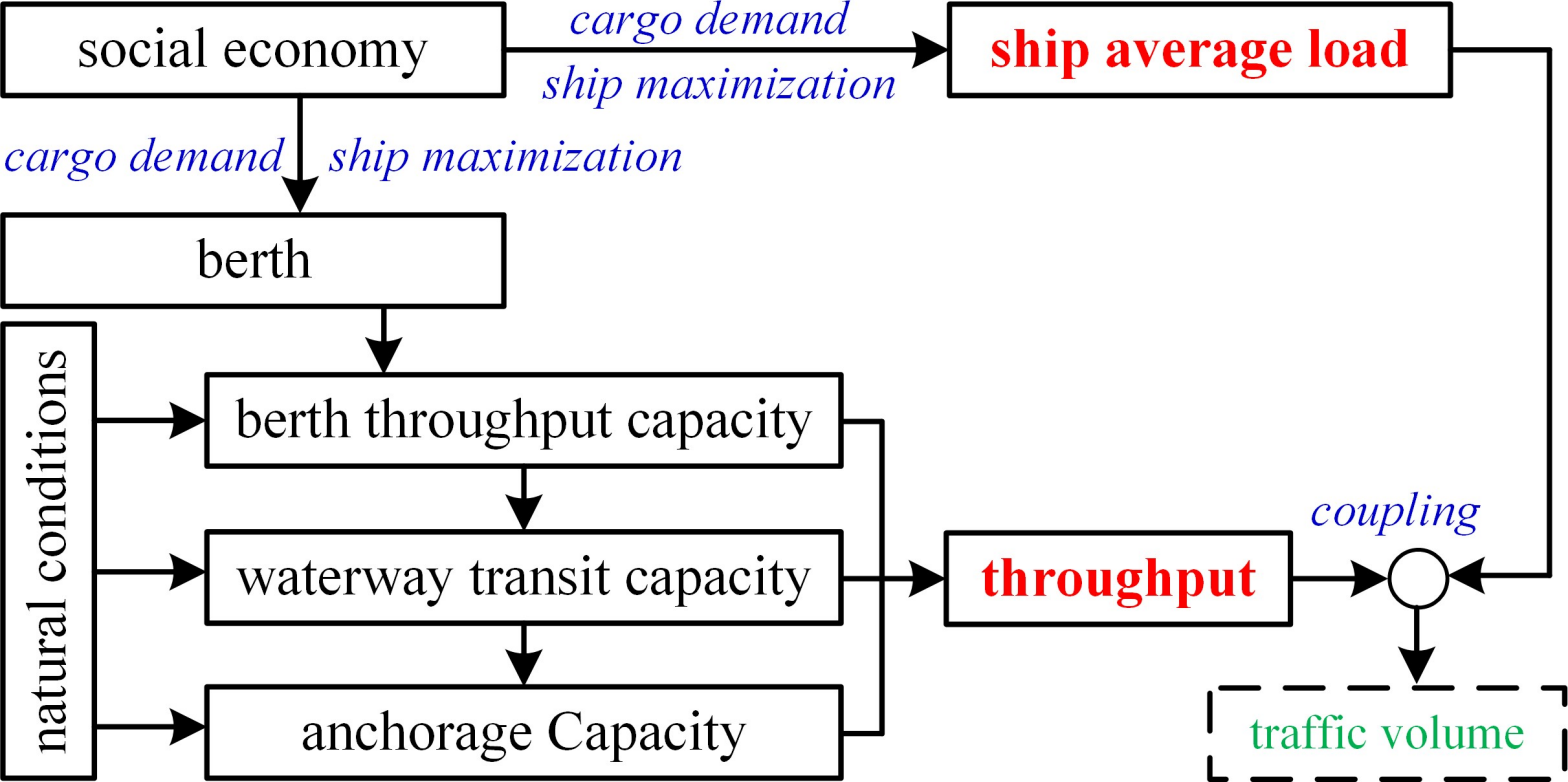
Impact Mechanism of the Factors Influencing Vessel Traffic Volume.

The main factors influencing vessel traffic volume include social economy, natural conditions, berth, route and anchorage, and natural conditions based on the hierarchical model. Fig 7 shows the impact mechanism of the factors influencing vessel traffic volume.

Fig 7 exhibits that the key factors influencing vessel traffic volume by port throughput and ship average load. The port throughput and ship average load can be obtained from the historical data which are therefore considered as quantitative indicators. The port throughput is a positive indicator, and the ship average load is a negative indicator.

S1 Table summaries number of container ships and container throughputs and container ship average loads of Port of Tianjin, china for period 2009–2014.

With data on container throughput, average load, and ships obtained for Port of Tianjin, Eqs (4) and (5) are employed to establish the standardized throughput and average load. Table 4 presents the standardized values.

**Table 4:** Standardized Throughput and Average Load of Port of Tianjin, China (2009–2014).

Year	2009	2010	2011	2012	2013	2014
Standardized throughput	0	0.245	0.545	0.679	0.811	1
Standardized average load	1	0.797	0.608	0.315	0.100	0

**Fig 8:**
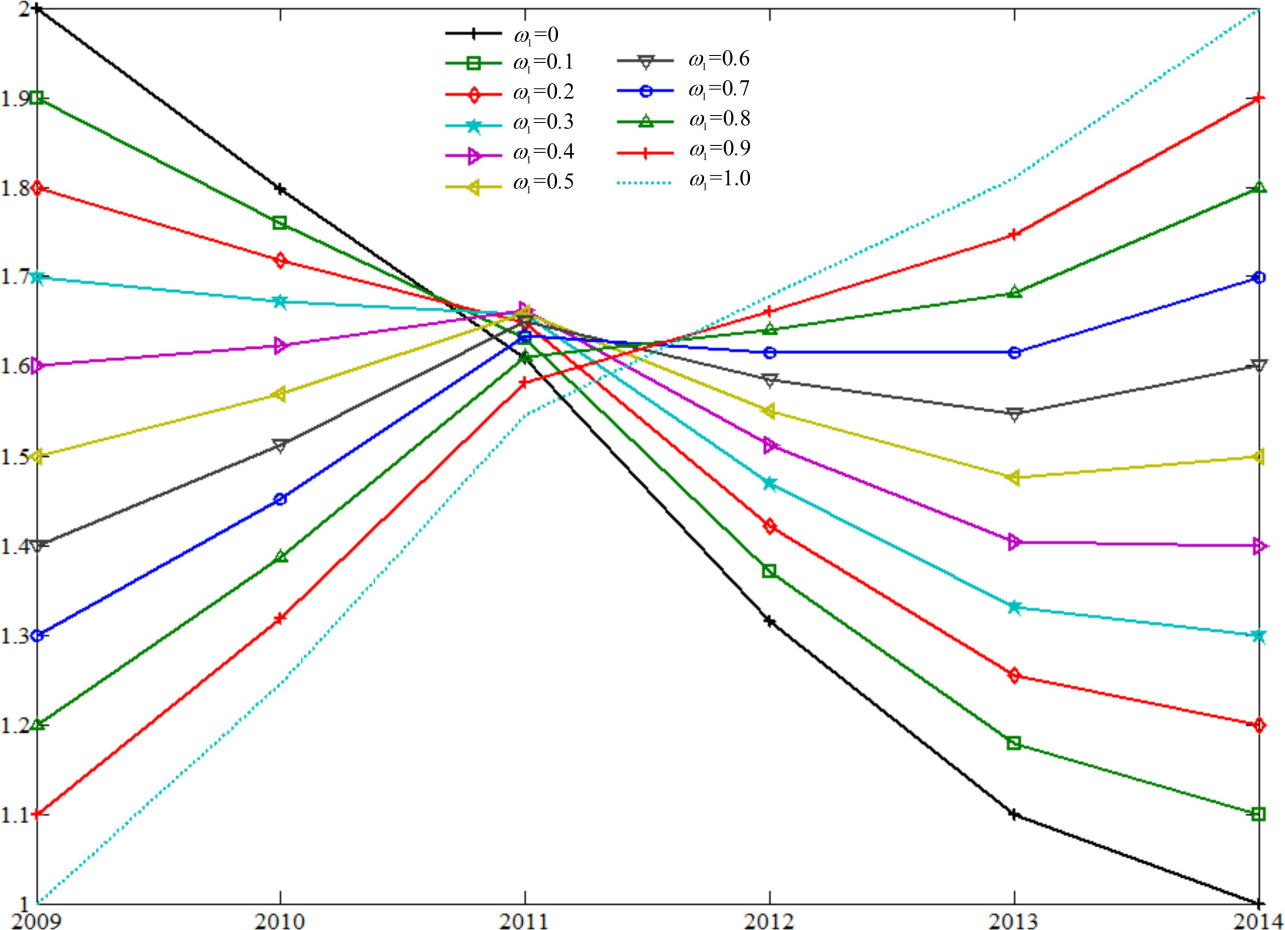
Coupling Coefficients of Different Weights of Port Throughput.

Next, Eq (7) is utilized to determine the coupling coefficients for standardized throughput and standardized average load. In particular, relative weight of the standardized throughput is varied from 0, 0.1, 0.2, 0.3, 0.4, 0.5, 0.6, 0.7, 0.8, 0.9, to 1 in the computation. Fig 8 shows coupling coefficients of different weights of the throughput.

Further, Statistical Product and Service Solutions (SPSS) software package for statistical analysis is implemented to establish statistical correlations between relative weight of the standardized throughput and number of container ships. Table 5 lists the results of the correlation analysis. Pearson’s correlation coefficient is at the maximum value of 0.996 when the relative weight of the throughput is set as 0.8, which is statistically significant at one percent significance level based on the two-tailed statistical test. For this reason, the relative weight of 0.8 is employed for throughput and the relative weight of the average load is set as 0.2 accordingly.

**Table 5:** Coupling Correlation and Two-tail Test Results.

Weight of throughput	0	0.1	0.2	0.3	0.4	0.5	0.6	0.7	**0.8**	0.9	1.0
Pearson correlation	-0.910*	-0.888*	-0.852*	-0.782	-0.600	0.065	0.879*	0.995**	0.996**	0.984**	0.972**
Sig. (2-tailed)	0.012	0.018	0.031	0.066	0.208	0.903	0.021	0.000	**0.000**	0.000	0.001

*, correlation is significant at the 0.05 level (two-tailed).

**, correlation is significant at the 0.01 level (two-tailed).

The coupling coefficient *μ*(*t*) as a function of standardized throughput and standardized average load is then given by Eq (9). Table 6 presents values of the coupling coefficient in different years.

$$\mu \left(t\right)=\left(1+0.8\cdot V_{1}^{+}\left(t\right)\right)\left(1+0.2\cdot V_{1}^{-}\left(t\right)\right)$$ (9)

**Table 6:** Coupling Coefficients from 2009 to 2014.

Year	2009	2010	2011	2012	2013	2014
Coupling coefficients	1.200	1.387	1.611	1.640	1.682	1.800

For further determining the coupling correlation coefficient *δ*(*t*), Eqs (10) and (11) are assumed:

$$Q\left(t\right)=q\cdot \delta \left(t\right)=q\cdot {\left(\mu \left(t\right)\right)}^{\frac{k}{2}}=q\cdot {\left(\mu \left(t\right)\right)}^{g}$$ (10)

$$Q\left(t\right)=q\cdot \delta \left(t\right)+c=q\cdot {\left(\mu \left(t\right)\right)}^{\frac{k}{2}}+c=q\cdot {\left(\mu \left(t\right)\right)}^{g}+c$$ (11)

where *Q*(*t*) is the number of containers (million TEU), *q* is the number of container ships, $V_{1}^{+}\left(t\right)$ is the standardized value of throughput, $V_{1}^{-}\left(t\right)$ is the standardized value of the average load, and *q*, *k*, *g*, and *c* are constants.

The data of container ships and coupling coefficients given in S1 Table and Table 6 is used to solve *q* and *k* in Eq (10) based on curve fitting using MATLAB R2014a. The optimal values of *q* and *k* are 4566 and 1.222 with 95 percent confidence bounds using Eq (10). The optimal values of *q*, *k* and *c* are 5466, 1.055, and -916 with 95 percent confidence bounds using Eq (11).

After obtaining values of the above constants in Eqs (10) and (11), the coupling correlation coefficients are computed. Table 7 lists the tested data of the number of container ships and the relative errors between field data and tested data.

**Table 7:** Validation Results of Coupling Model.

Year	2009	2010	2011	2012	2013	2014
Coupling correlation coefficients using Eq (10)	1.118	1.221	1.338	1.353	1.374	1.432
Tested data of container ships using Eq (10)	5104	5576	6110	6179	6274	6539
Coupling correlation coefficients using Eq (11)	1.101	1.188	1.286	1.298	1.316	1.364
Tested data of container ships by using Eq (11)	5102	5579	6113	6181	6275	6537
Field ship number	5100	5576	6184	6150	6213	6557
Relative errors of Eq (10)	0.08%	0.00%	**1.20%**	0.47%	0.98%	0.27%
Relative errors of Eq (11)	0.04%	0.06%	**1.14%**	0.50%	1.00%	0.31%

## Discussion

As shown in Table 7, results from the coupling model and the case study demonstrate that the mean relative errors of Eqs (10) and (11) are 0.60 percent and 0.61 percent, respectively, and the highest relative errors calculated using the two equations are 1.20 percent and 1.14 percent correspondingly. The relative errors are rather small, and the coupling model is accredited to reveal the impact mechanism of the factors on vessel traffic flow. The findings are summarized as follows.

(1) The hierarchical model reveals the impact mechanism and the relationships of factors influencing vessel traffic flow. The model identifies factors at the top level that affect vessel traffic flow directly, and factors at the lowest level affect not only vessel traffic flow but also other factors. Hence, a quick and effective way of improving vessel traffic flow is improving the factors at the top level. An effective means of improving vessel traffic flow is to increase the performance levels of the constituent factors. The most effective way of improving vessel traffic flow is to enhance multi-levels factors with duly cognizance of the impact mechanism of those factors.

(2) The validation results of the proposed coupling model show that they are highly consistent with the field data used for cross comparisons. This provides evidence that the calculated weights and constants that are further used for computing coupling correlation coefficients are adequate.

(3) In the process of deriving the coupling correlation coefficient of vessel traffic volume, relative weights of the indicators comprised of standardized throughput and standardized average load. It reveals that the relative importance is higher for port throughput than the average load of the ships.

The above findings suggest that the proposed models would help propose measures to improve vessel traffic flow and navigation safety.

## Conclusion

This study introduces a hierarchical model and a coupling model of factors that influence vessel traffic flow. The hierarchical structure of the influencing factors is analyzed, along with development and application of the hierarchical model. The performance of the coupling model is validated based on the data collected from Port of Tianjin, China by analyzing vessel traffic volume. The hierarchical model of the factors that influence vessel traffic flow shows the relationships of the influencing factors intuitively and clearly using a five-level hierarchical system. Effective measures for enhancing vessel traffic flow can be proposed based on the hierarchical model. Moreover, the proposed coupling model reveals the coupling effect of influencing factors by identifying quantitative indicators to a designated issue of vessel traffic flow based on the impact mechanism of the influencing factors. The findings of model validation give evidence that the coupling model is capable of analyzing vessel traffic volume.

In future studies, measures for improving vessel traffic flow should be proposed based on influencing factors identified in the hierarchical model. Also, effectiveness of such measures should be analyzed. In addition, other elements of vessel traffic flow such as direction, width, density, and speed should be included into the coupling model for validation, and the coupling model should be used to predict the changes in the issues based on the multi-dimensional historical data.

## Supporting information

S1 Table Container shipments of Port of Tianjin, China (2009–2014). S1 Table summaries number of container ships and container throughputs and container ship average loads of Port of Tianjin, china for period 2009–2014.
(DOCX)
