# Supplementary material for: Hierarchical and coupling model of factors influencing vessel traffic flow
Source: PLoS One. 2017 Apr 17;12(4):e0175840. doi: 10.1371/journal.pone.0175840 (PMC5393871; doi:10.1371/journal.pone.0175840)
Supplement: S1 Table — summaries number of container ships and container throughputs and container ship average loads of Port of Tianjin, china for period 2009–2014. (DOCX) [file pone.0175840.s001.docx]

**S1 Table. Container shipments of Port of Tianjin, China (2009-2014).**

| Year | 2009 | 2010 | 2011 | 2012 | 2013 | 2014 |
| --- | --- | --- | --- | --- | --- | --- |
| Number of container ships | 5100 | 5576 | 6184 | 6150 | 6213 | 6557 |
| Container throughputs (million TEU) | 8.70 | 10.00 | 11.59 | 12.30 | 13.00 | 14.00 |
| Container ship average loads (TEU) | 1706 | 1793 | 1874 | 2000 | 2092 | 2135 |
